# Computational Challenges in Tissue Engineering for the Spine

**DOI:** 10.3390/bioengineering8020025

**Published:** 2021-02-15

**Authors:** André P. G. Castro

**Affiliations:** IDMEC, Instituto Superior Técnico, Universidade de Lisboa, 1049-001 Lisboa, Portugal; andre.castro@tecnico.ulisboa.pt

**Keywords:** spine, intervertebral disc, vertebrae, tissue engineering, finite element modelling, biomechanics

## Abstract

This paper deals with a brief review of the recent developments in computational modelling applied to innovative treatments of spine diseases. Additionally, it provides a perspective on the research directions expected for the forthcoming years. The spine is composed of distinct and complex tissues that require specific modelling approaches. With the advent of additive manufacturing and increasing computational power, patient-specific treatments have moved from being a research trend to a reality in clinical practice, but there are many issues to be addressed before such approaches become universal. Here, it is identified that the major setback resides in validation of these computational techniques prior to approval by regulatory agencies. Nevertheless, there are very promising indicators in terms of optimised scaffold modelling for both disc arthroplasty and vertebroplasty, powered by a decisive contribution from imaging methods.

## 1. Introduction

Lumbar spine conditions related to degeneration are a serious source of disability worldwide, as they affect a large portion of the population. This results in high care costs for therapy and treatment, especially in Western societies. Currently, this problem is one of the major reasons for work absenteeism and productivity decrease, denoting itself to be an alert for society [1,2]. Recent statistics from the current decade point out that 70% to 85% of the Western population has, or is expected to have at some point, a record of low back pain (LBP), the global prevalence of chronic LBP being estimated between 7% and 9% [3,4].

The high frequency of these conditions can be explained by the ageing of the population and by the prominence of sedentary lifestyles, but its actual roots are far from being fully understood [5]. Intervertebral disc (IVD) degeneration or ligament hypertrophy, which in the most severe cases can greatly impact people’s quality of life, are among the known risk factors [2,6].

The IVD works with vertebral bodies (VB), ligaments and muscles to provide flexibility, shock absorbance and motion to the spine [7]. It has three components: (i) nucleus pulposus (NP), a highly hydrated biphasic material with dispersed collagen fibres; (ii) annulus fibrosus (AF), composed of successive lamellae of highly anisotropic collagen fibres, responsible for containing the NP; and (iii) cartilaginous endplate (CEP), a thin layer of hyaline cartilage, responsible for most of the nutrient exchange [8,9,10]. The complexity of each component and their connection is a challenge for both healthiness and degeneration studies. Each VB–IVD–VB complex is denoted as a functional spinal unit (FSU), the basic unit of the spine [11,12].

Furthermore, ligaments and muscles play an important role in stability as well as in motion proprioception. There are seven major ligaments constraining the spine components and limiting the range of motion (ROM) in all directions. Due to their elasticity, they passively help return the spine back to the neutral position [13,14]. These ligaments of the spine are the ligamentum flavum (LF), attached between the lamina of each VB; the anterior longitudinal ligament (ALL); the posterior longitudinal ligament (PLL), which works with the ALL along the consecutive VB; the intertransverse ligament (ITL), which connects the transverse processes; the interspinous ligament (ISL), which connects the opposing edges of the spinous processes; the supraspinous ligament (SSL), which connects the peaks of the spinous processes; and the capsular ligament (CL), which connects the circumferences of the joining articular facet joints [15,16]. The first three ligaments allow for flexion and extension of the spine, while keeping the bony parts in their correct alignment. The remaining four ligaments connect all the posterior elements of the VB [13,17].

Degenerative spine diseases (DSD) associated with LBP include degenerative disc disease (DDD), spondylolisthesis (wherein a vertebra suffers a displacement relative to the adjacent one) and stenosis (narrowing of the spinal canal) [18,19]. A lack of widely accepted explanatory models limits the understanding of such diseases, retarding the development of effective therapies [20,21]. The most common treatments of DSD are either conservative (no surgery) or surgical (invasive). However, these methods may act only to relieve symptoms; further complications frequently arise from massively invasive procedures, such as interbody fusion, disc arthroplasty or vertebroplasty [22,23,24,25,26]. Therefore, minimally invasive surgical approaches, based on tissue engineering (TE) methods, should be applied, whenever possible, to recover healthy functionality of the spine [27,28,29]. Additionally, to better diagnose and treat spinal instability, as well as to create models to support clinical decisions and surgical procedures, it is of utmost relevance to understand the kinematics and constitutive mechanics of the spine. Since in vitro and in vivo studies have some inherent limitations, computational models are useful tools in the investigation of these issues [30,31].

Therefore, this brief review intends to demonstrate the growing importance of computational methods in the study of the healthy, degenerated and implanted human spine, as well as to identify the next generation of simulation-based TE studies towards the development of increasingly adequate treatment solutions for DSD and LBP.

## 2. Computational Biomechanics

Since the first application of the finite element (FE) method in biomechanics by Brekelmans et al. in 1972 [32] and the first reported spine model developed by Belytschko et al. in 1974 [33], the number of studies on the human spine without resorting to animal and cadaveric experiments has increased significantly [34]. This application has countless benefits to health and biomedical research. As the human spine is a complex system, the intrinsic easiness with FE modelling in changing the geometry, constitutive laws, material properties and boundary conditions can lead to many important advances regarding spinal health, diseases, degeneration, trauma, surgery procedures and spinal instrumentation. Other computational methods, such as multibody systems modelling (usually dedicated to movement studies), can also be linked with FE modelling to establish multiscale approaches to spine biomechanics, providing macromechanical movement data (e.g., load, pressure or velocity) as simulation inputs [35,36].

In fact, FE modelling has already led to relevant conclusions and supported many pre-clinical studies, namely in what concerns to IVD behaviour or implant development [8,37,38,39,40]. Additionally, it has also motivated the development of new computational tools (imaging reconstruction methods, FE solvers or even kinematics simulators) that have later led to wider achievements in biomechanics and tissue engineering [41,42,43,44,45,46,47].

### 2.1. Macromechanical Level

From the works of Breau et al. in 1991 [48] or Chen et al. in 2001 [49], in which FE models of the lumbar spine were built from computed tomography (CT) scans, a more accurate perspective of the spine components was potentiated. The first group developed an L1–S1 model, including the seven relevant spine ligaments (LF, ALL, PLL, ITL, ISL, SSL and CL) plus the iliolumbar and the fascia, simulated by uniaxial elements. The AF was modelled as a ground substance with collagenous fibres embedded, while the NP was represented as an inviscid fluid. The fact joints were designed as a general moving contact problem [48]. The second group included a similar FSU modelling approach, with the seven ligaments represented by cable elements, along with the fibres of the AF. The facet articulations were modelled as 3D contact elements. This model was employed in simulating a lumbar fusion intervention, in which the IVD of the affected level was replaced by an interbody bone graft. It was possible to study the ROM and the stress distribution on the adjacent discs after surgery, which would not be directly possible without these numerical methods [49].

**Figure 1:**
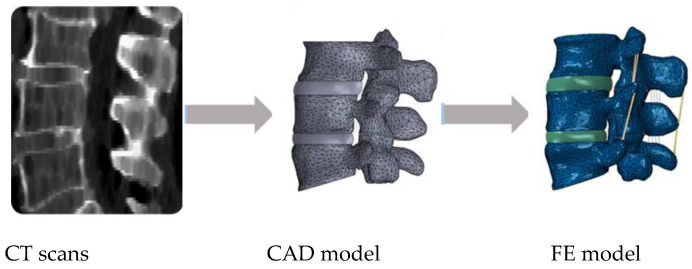
Example of a common pathway for patient-specific finite element (FE) modelling of the spine, going from medical imaging to the final FE model.

Figure 1 shows a common patient-specific pathway for 3D model reconstruction from imaging examinations (CT scans), considering the design of the IVD on a given computer aided design (CAD) software and then the inclusion of the ligaments within the FE modelling software.

More recent studies were conducted to investigate new possible designs for spine implants. Choi et al. in 2016 [50] studied the biomechanical effects in surgical and adjacent FSUs of a newly interspinous process compressor and compared it with the traditional pedicle screw fixation. The different FE models of the lumbar spine were evaluated in terms of the ROM, facet contact forces and stress in the adjacent IVD. Guo and Yin in 2019 [51] proposed a new device interspinous stabilisation process using topology optimisation, which is a very important numerical technique to achieve better designs and more adequate material properties for biomedical devices [52,53]. A new device with reduced volume was fabricated from their FE modelling strategy, and the results showed that the innovative device provided stability in all motion directions at the affected segment, while decreasing the stress of the implant structure [51]. Lu and Lu in 2019 [54] built an L3–L5 FE model comprising the NP modelled as a linear elastic fluid, the AF represented by a ground substance with layers of diagonal fibres of varying strength and the seven major ligaments. Different interventions were simulated at the L4–L5 level, leading to the conclusion that lumbar interbody fusion techniques permitted greater stability, having shown a smaller ROM and stress peaks on the posterior instrumentation than posterolateral fusion. Demir et al. in 2020 [40] compared three different hybrid stabilisation systems (combination of dynamic and static fixation), starting from an L1–L5 FE model from CT scans. Hybrid stabilisation has been a focus of discussion as regards lumbar interbody fusion, and this study was able to demonstrate that the movement supported by the dynamic fixation in the adjacent level could reduce fracture risk and the overall intradiscal pressure (IDP). Such insight into tissue behaviour and mechanical responses would also not be possible with other in vitro or ex vivo techniques [8,54]. Zhou et al. in 2020 [55] combined in vivo imaging with FE simulation to evaluate the dynamics of adjacent-level degeneration after interbody fusion, creating a framework for pre- and post-operative assessment of the patients. This study is important to show the way for simulation integration in clinical practice.

### 2.2. Tissue Level

The development of implants or new surgical approaches is definitely within the top priorities for human spine studies. However, there are still questions to be answered as regards the biomechanics and mechanobiology of native spine components. Experimental characterisation and numerical modelling of the IVD have been a challenge throughout the years, particularly as regards its multiphasic charge-driven behaviour and the anisotropic behaviour of its fibres [9,56,57,58,59,60]. CT scans are one of the sources of imaging data, given their high resolution, although magnetic resonance imaging (MRI) is more accurate for IVD degeneration detection. X-ray scans are more limited due to their 2D nature, but they are also employed as they permit less radiation exposure than CT equipment when considering in vivo evaluations [44,61,62,63].

Table 1 lists the most common constitutive models and data sources for different FSU tissues. It must be highlighted that despite the complex morphology of bone, cortical bone is usually modelled as linear elastic and isotropic; the local trabeculae of trabecular bone are markedly anisotropic, but such behaviour is not frequently taken into consideration in FSU modelling.

Additionally, known DDD triggers such as osmotic pressure variation, CEP calcification, NP internal cracks or AF bulging are directly associated with intricate constitutive properties [27,68]. Consequently, the characterisation of FSU tissues (not only the IVD but also the VB, muscles and ligaments) before and after initiation of the degenerative process is the most accurate strategy to tackle the alterations associated with DDD and find potential treatments [44,69,70,71], i.e., it is not realistic to study a DDD symptom such as IVD height decrease or dehydration without analysing each adjacent component.

The work of Schroeder et al. in 2010 [64] was one of the first and most complete approaches to the real mechanical behaviour of the IVD by implementing a 3D osmo-poro-visco-hyper-elastic FE model. In this study, the behaviour of the IVD was simulated as a function of NP and AF tissue biochemical composition, organisation and constituent properties, bridging chemistry, biology and physics. Fluid flow or the AF bulging response was consistent with experimental studies, demonstrating that a comprehensive approach is necessary to understand the overall IVD response. The idea of studying the influence of each specific parameter in order to distinguish between native and degenerated conditions was then followed in many other works [8,72,73,74].

The most recent generation of IVD/FSU studies is focused on patient-specific modelling, i.e., to associate what is known about IVD or VB biomechanics with the specific characteristics of each patient [75]. However, this strategy will only be effective with large databases of cases, provided that such databases contain complete medical data: top-quality medical imaging examinations are not enough, as the clinical background of the patient is also necessary to achieve a meaningful patient-specific FE model of the spine [76]. Fattor et al. in 2016 [77] employed CT scans to tailor total disc replacement devices and hence ensure correct geometrical fitting. The study considered not only pre-operative examinations but also follow-up post-operative data to establish a complete evaluation of the expected device performance and intraoperative alignment.

The works of Lavecchia et al. in 2018 [47] or Bojairami et al. in 2020 [78] show dedicated tools for the generation of geometrically accurate models for patient-specific FE studies, based on either age-height correlation for a given population or imaging examinations. This eliminates the need for a full 3D reconstruction from imaging, while ensuring good-quality FE meshes. Still, it must be highlighted that geometry is only one of the vectors to be considered in patient-specific spine modelling. The work of van Rijsbergen et al. in 2018 [79] is one of the most complete approaches to understanding how to go from imaging to actual clinical decision support. The developed framework to predict treatment outcomes is supported in high-level numerical modelling of FSU tissues, from mechanoregulation of the IVD environment to adjacent bone remodelling.

A different trend of FE-based studies is to emphasise on the modelling of specific FSU tissues, namely the cortical/trabecular layers of the VB [62,66,80,81], the layered organisation of the AF [58,65,82,83] or the fluid exchange and osmotic swelling properties of the NP [84,85,86,87]. These (and other) studies are contributing to identifying the complexity of FSU tissues and to understanding their interaction, ranging from vertebroplasty support to the importance of the AF criss-cross angles in the herniation process, despite the frequent simplifications on the modelling of surrounding tissues (i.e., the FSU components that are not the focus of such study).

The common ground is that although numerical methods are increasingly established for tissue characterisation, the experimental support (through cadaveric samples or in vivo imaging) are more important than ever to validate these achievements and to obtain the approval by regulatory agencies for their use in clinical practice [47,56,88,89,90].

## 3. Challenges

Similar to tissue characterisation, FE modelling is now an essential tool for the development of TE solutions. This review intends to look at the application of FE modelling in TE studies, instead of looking at the multiple TE works dedicated to human spine tissue treatment or substitution.

TE substitutes to the IVD must mimic its complex environment and the interactions between the major components under native and degenerated conditions, which leads to a considerable number of design requisites. The IVD is an osmo-poro-hyper-viscoelastic organ, with anisotropic fibre reinforcement, so its mechanical functionality in terms of load support and flexibility needs to be ensured in arthroplasty or partial regeneration (e.g., NP replacement with hydrogel) [29,91,92].

The work of Silva et al. in 2005 [93] was one of the first to include FE simulation in the evaluation of a TE hydrogel for IVD replacement, the hydrogel being supported by an annular ring, i.e., mimicking the native NP–AF structure. Poro-elastic simulation allowed for the verification of stress patterns generated by this substitute under physiological fluid exchange. Strange et al. in 2010 [94] developed an FE model to assess the behaviour of an elastomeric NP replacement, assuming that such implant would have different mechanical properties than the native IVD, namely a different compressive stiffness. The focus was again on the stress distribution patterns: the NP natively distributes the pressure to the AF, which is then responsible for supporting the circadian variation. Different nucleotomy procedures/implant sizes were studied towards achieving the most appropriate solution, before advancing to actual implant production and approval. Schmidt et al. in 2014 [95] also investigated the effect of geometry/volumetric properties of an NP substitute, but here mimicking the poro-elastic medium of the IVD and performing an overall assessment of the implant behaviour, i.e., evaluating its effects on the adjacent FSU components. Kang et al. in 2013 [96] and Zhang et al. in 2018 [97] evaluated the design or porosity of interbody fusion cages towards achieving an optimal solution for each specific spinal level. Lim et al. in 2019 [98] addressed the issue of cage subsidence by combining additive manufacturing techniques and FE simulation to compare the behaviour of solid vs. porous devices built with PEEK–titanium composites, highlighting the biomechanical advantages of using a porous structure that could be built only with titanium. Their study is important to look at the multiple possibilities of computational simulations in TE, both for design and/or for testing of solutions.

Still, most of these studies have focussed on achieving a coherent geometry to the intervertebral space available after discectomy or nucleotomy, which is of upmost importance to ensure soft integration of the implant and an appropriate response of the surrounding tissues. The step forward from generic to dedicated solutions is another important aspect of the most recent computationally based TE approaches as regards structural optimisation methods [52,96]. The advances in additive manufacturing have potentiated the applicability of these tailored solutions. As seen above, the other level of research is the material properties of the substitute and their configuration, which will also be pivotal for the success of the procedure. Additive manufacturing has a main role here, as the use of biocompatible materials in these technologies is increasingly frequent [38,99]. However, as happens with the modelling (and subsequent substitution) of every biological tissue, the dependence of the constitutive material parameters on the patient’s age, physical condition and clinical history has to be addressed in such TE strategies [100,101,102]. This means that it may not be a good strategy to replace an aged IVD with a TE substitute based on the characteristics of a young and barely degenerated IVD, which will most likely induce a different biomechanical response. If the option is for interbody fusion instead of arthroplasty, the replacement cage should be adapted to the environment of the affected level and not to its native geometry/material properties as regards rehabilitating the correct sagittal balance for that specific patient/situation [103,104].

In other words, a given device may have been modelled according to the best practices and produced with expensive techniques and materials, but it may still fail if careful pre-analysis of the individual situation is not performed. FE modelling then appears as the key technique to pre-screen the mechanobiological environment prior to the clinical decision, despite the challenging conditions to achieve significant simulations.

## 4. Conclusions

To conclude, it has been demonstrated here that FE modelling is nowadays an essential tool to pre-screen the specificities of a patient suffering from LBP, particularly the targeted tissues. It is also important to evaluate the potential TE solutions to be employed. Still, with growing computational power and developing imaging methods, the contribution of medical imaging examinations is decisive, along with the also extremely important contribution of clinical essays. Therefore, the major challenge in computational modelling for TE solutions to tackle DSD is to achieve comprehensive strategies that include the tissue properties and the functionality target. TE developments still have a way to go before full clinical practice implementation and fast regulatory approval, but the association of FE modelling is definitely the most promising approach to achieve successful patient-specific solutions.

## Figures and Tables

**Table 1:** Common constitutive modelling options and preferential imaging methods to characterise functional spinal unit (FSU) tissues.

**Component**	**Constitutive Model**	**Imaging Method**
NP	Hyper-elastic, poro-elastic with osmotic swelling [41,64]	MRI
AF	Hyper-elastic, anisotropic (fibre reinforcement), poro-elastic with osmotic swelling [41,65]	MRI
CEP	Hyper-elastic, poro-elastic [41]	MRI
Cortical bone	Linear elastic, orthotropic [66]	CT
Trabecular bone	Hyper-elastic, anisotropic [66]	CT
Ligaments	Non-linear, tension-only [17]	MRI
Muscles	Non-linear [67]	MRI

MRI: Magnetic resonance imaging; CT: Computed tomography.
